# Single-particle excitations in the uniform electron gas by diagrammatic Monte Carlo

**DOI:** 10.1038/s41598-022-06188-6

**Published:** 2022-02-10

**Authors:** Kristjan Haule, Kun Chen

**Affiliations:** 1 Department of Physics and Astronomy, Rutgers University, Piscataway, NJ 08854 USA; 2 Center for Computational Quantum Physics, Flatiron Institute, The Flatiron Institute is a division of the Simons Foundation, 162, 5th Avenue, New York, NY 10010 USA

**Keywords:** Electronic properties and materials, Electronic properties and materials

## Abstract

We calculate the single-particle excitation spectrum and the Landau liquid parameters for the archetypal model of solids, the three-dimensional uniform electron gas, with the variational diagrammatic Monte Carlo method, which gives numerically controlled results without systematic error. In the metallic range of density, we establish benchmark values for the wave-function renormalization factor *Z*, the effective mass $m^*/m$, and the Landau parameters $F_0^s$ and $F_0^a$ with unprecedented accuracy, and we resolve the long-standing puzzle of non-monotonic dependence of mass on density. We also exclude the possibility that experimentally measured large reduction of bandwidth in Na metal can originate from the charge and spin fluctuations contained in the model of the uniform electron gas.

## Introduction

The uniform electron gas (UEG) is the most fundamental model for understanding the electronic properties of metallic materials. The ground-state properties of the model have been very precisely calculated by quantum Monte Carlo methods^1^, and this allowed one to build approximate density functionals^2,3^, which are at the heart of the ab-initio approaches in material science and modern theory-driven materials design. The knowledge of the low energy excitations of the same model remain challenging to evaluate accurately^4–10^, even though such calculations are important for building more sophisticated density functionals^11–13^, and these excitations are directly measured in experiments on simple metals, such as alkaline materials. Some aspects of the excitation spectra, such as the quasiparticle renormalization amplitude, were recently determined by extention of the variational Monte Carlo method in Ref.^14^, which turn out to be in very good agreement with our current results.

In the metallic regime, the low-energy properties of the electron liquid are dominated by the long-lived quasiparticles near the Fermi surface, and their dynamics is described by a handful of the Fermi liquid parameters. These parameters completely characterize the low energy excitation spectra of the metallic state. Unfortunately, they are very challenging to calculate by a first principle approach, therefore they are usually treated as phenomenological parameters requiring input from experiments.

Here we develop an extension of the recently introduced variational diagrammatic Monte Carlo (VDMC) method^15^, which fills this void, and allows us to determine the single-particle excitations of UEG with unprecedented accuracy. In this letter, we calculate the single-particle excitation spectra, and in particular, we give controlled values of the wave-function renormalization factor *Z*, the quasiparticle effective mass ratio $m^*/m$ and also the Landau Fermi liquid parameters $F_0^a$ and $F_0^s$. Our computed values are free of systematic error, and their uncertainty is mainly controlled by the statistical error, and hence our established value can be used as a precise benchmark for new method development. Moreover, these precise Fermi liquid parameters are also useful for building more sophisticated density functionals. Finally, the method we develop here can be used to solve more sophisticated models, and can also be used in the ab-initio framework on models of realistic materials, a development which is currently underway^16^.

## Results

### The Feynman expansion algorithm

The VDMC method^15^ is a flavor of diagrammatic Monte Carlo method (DMC)^17–24^, which samples high-order Feynman diagrams with a Monte Carlo importance sampling. The novelty of VDMC is two-fold: (1) it optimizes the starting point of the perturbative expansion in such a way that the expansion converges very rapidly with the increasing perturbation order. (2) it efficiently combines an exponentially large number of Feynman diagrams, which mostly cancel among themselves due to alternating fermionic sign so that the groups of diagrams can be efficiently sampled with the Monte Carlo importance sampling hence avoiding the explosion of statistical error with perturbative order.

In Ref. ^15^ we computed the spin and the charge response functions of the UEG model with VDMC by evaluating the Feynman diagrams for the polarization function. A similar type of Feynman expansion in terms of non-interacting single-particle Green’s function, and statically screened Coulomb interaction does not converge rapidly enough to establish a reliable infinite order result, hence we here develop an alternative approach.

**Figure 1:**
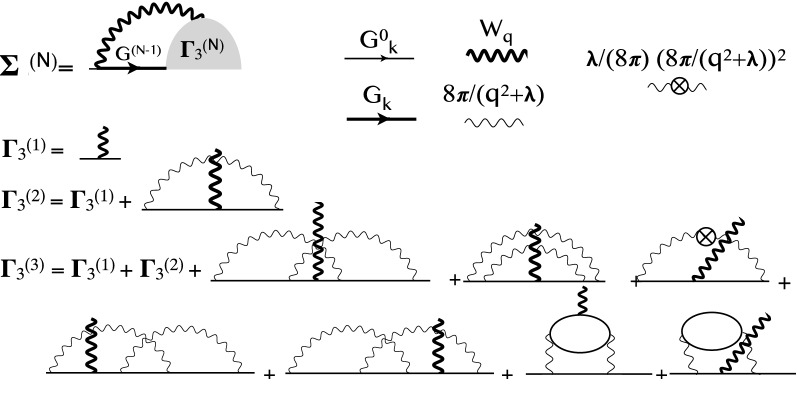
Feynman diagrams for the self-energy in terms of the three leg vertex $\Gamma _3$, which is expanded in bare series in terms of $G_{\mathbf {k}}^0$ and partially screened interaction $v_{\mathbf {q}}=\frac{8\pi }{q^2+\lambda }$ and counter-terms $(\frac{\lambda }{8\pi })^N(\frac{8\pi }{q^2+\lambda })^{N+1}$. The dressed $W_{\mathbf {q}}$ was computed in Ref. ^15^, and $G^{(N-1)}=((G^0)^{-1}-\Sigma ^{(N-1)})^{-1}$ is determined from previous order $\Sigma ^{(N-1)}$, which is stored and reused.

In this work, we show that extremely rapid convergence with perturbation order can be achieved by using a Hedin-type equation, in which we first compute the numerically exact screened interaction $W_{\mathbf {q}}$ (previously developed in Ref. ^15^), and we then expand only the three-point vertex function $\Gamma _3$ in powers of the bare electron propagator $G^0_{\mathbf {k}}$, and statically screened interaction $v_q(\lambda )$, with proper counter terms defined in the Method section. Here the screened Coulomb interaction $v_q(\lambda )$ has a Yukawa form, characterized by the inverse screening length $\lambda$. This screening parameter has to be determined by the principle of minimal sensitivity in order to achieve rapid convergence of the perturbative series, so that the extrapolation to infinite order is possible. Figure 1 shows the sketch of the corresponding Feynman diagrams up to the third order. Below we apply the algorithm to the UEG model, although the method is completely general and could as well be carried out for realistic material in the ab-initio framework.

### The single particle excitations

**Figure 2:**
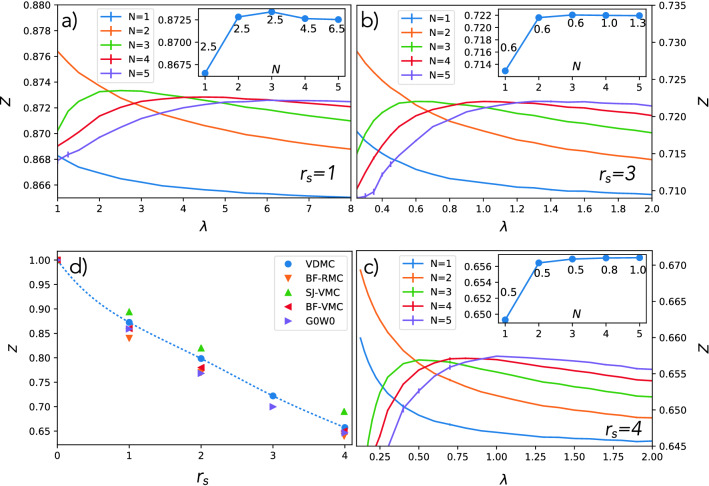
The wave-function renormalization factor *Z* versus screening parameter $\lambda$ for various perturbation orders $N=1...5$ and for $r_s=1, 2, 3$ and 4. The insets show the convergence of *Z* with perturbation order *N* when its value is taken at the extremal $\lambda$. The numbers next to each point show the value of $\lambda$ used for each calculated point. Panel d) compares current VDMC results with prior Monte Carlo results from Ref.^14^ and G0W0 from Ref.^25^.

We first present the single-particle excitation spectral results. Figure 2a–c show how the wave-function renormalization factor *Z* depends on the screening parameter $\lambda$ in our theory. To determine the optimized parameter $\lambda$, we scan $Z(\lambda )$ for each $r_s$, and determine it with the principle of minimal sensitivity. For efficiency, we here sample the self-energy only at the Fermi wave vector $k_F$ and at the two lowest Matsubara frequencies, which is sufficient to determine *Z*. We notice that for the first two orders, no counter term in the parameter $\lambda$ occurs, therefore the curve $Z(\lambda )$ displayed in Fig. 2 does not have extremum, while all higher-order terms have a well-defined maximum, which broadens and develops into a broad plateau with increasing order. The insets of Fig. 2a–c show optimized *Z* versus perturbation order, where the first two orders are evaluated at the optimal $\lambda$ of the third order, and for later orders, we take the value in the maximum. We also display the value of $\lambda$ used at each order. From Fig. 2 it is apparent that beyond order three the rate of convergence to limiting value of *Z* is extremely fast, and therefore we can confidently determine the first three digits of *Z*. The values and the estimated error-bar from the extrapolation and statistical errors are shown in Table 1.

**Table 1:** Landau liquid parameters: The wave-function renormalization factor *Z*, effective mass $m^*/m$, and the Landau parameters $F_0^a$, $F_0^s$ for various values of the density parameter $r_s$ with the estimated error.

$r_s$	*Z*	$m^*/m$	$F_0^a$	$F_0^s$
1	0.8725(2)	0.955(1)	$-$ 0.171(1)	$-$ 0.209(5)
2	0.7984(2)	0.943(3)	$-$ 0.271(2)	$-$ 0.39(1)
3	0.7219(2)	0.965(3)	$-$ 0.329(3)	$-$ 0.56(1)
4	0.6571(2)	0.996(3)	$-$ 0.368(4)	$-$ 0.83(2)

In Fig. 2d we compare our computed $Z(r_s)$ with the previous best available estimates, obtained by various flavors of Monte Carlo (MC) methods, which are reproduced from Ref.^14^. Note that all these published MC methods rely on fixed node approximation and the thermodynamic limit extrapolation, hence they have an inherent systematic error, nevertheless they turn out to be in very good agreement with current VDMC results.  The VDMC method has well controlled numerical error which can be made very small. It originates from the statistical error due to MC sampling, and the error due to extrapolation from finite order to infinite order of expansion, which is well behaved in the metallic regime r_s_≤4. We note that VDMC has no systematic error. We notice that previous MC results are broadly consistent with our results, with SJ-VMC method predicting slightly too large and BF-VMC and BF-RMC slightly too small value. It is also well known that G0W0 predicts quite accurate *Z* values, however, we can now confidently claim that in the range of metallic densities, G0W0 consistently underestimates *Z*.

**Figure 3:**
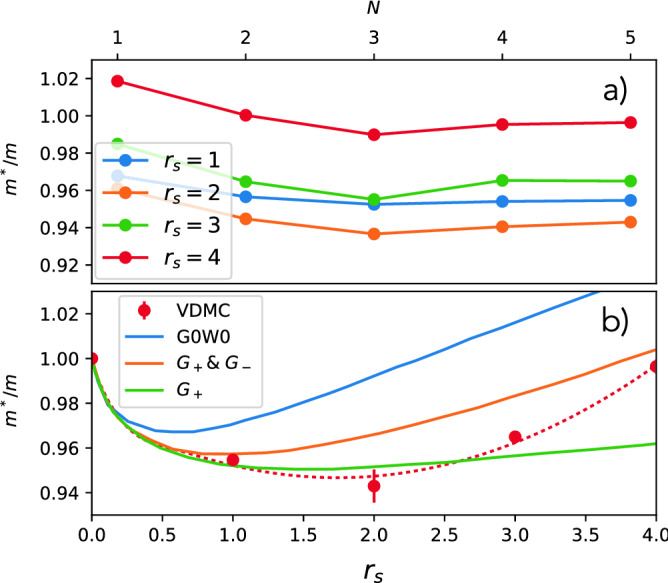
Electron effective mass: The upper panel shows our calculated effective mass versus perturbation order for $r_s=1-4$ (the statistical error-bar is smaller than the size of the symbols). The lower panel compares the $r_s$ dependence of the effective mass of this work (VDMC) with the prior analytic and numeric work from Ref. ^26^.

Once the extremal value of $\lambda$ is determined, we compute the entire momentum and frequency dependence of the self-energy, which allows us to determine also the momentum derivative of the self-energy, and hence the effective mass of the electron through the relation

1 $$\begin{aligned} \frac{m}{m^*} = Z \left( 1 + \frac{m}{k_F}\frac{d\Sigma (k_F,\omega =0)}{dk}\right) \end{aligned}$$

The convergence of the effective mass ratio $m^*/m$ with perturbation order is shown in Fig. 3a, and its dependence on $r_s$ is displayed in Fig. 3b.

The dependence of the effective mass $$m^*/m$$ on $$r_s$$ has been controversial for many decades. Some theories predict that the ratio is monotonically decreasing with increasing $$r_s$$^7,27^, while others predict the existence of a turning point $$r_s^*$$^26,28–31^ at which the trend is reversed. Our controlled results confirm the correctness of the later theories. Furthermore, we compare our controlled VDMC results with previous best estimates, which are based on the theory of many-body local field factors^26^. This theory includes vertex corrections associated with charge and spin fluctuations, extracted from available Monte Carlo data. We notice that G0W0 overestimates the effective mass in the entire range of metallic densities. The density fluctuations beyond RPA are included in theory with $$G_+$$ local field corrections, which reduce the mass substantially and bring it very close to our VDMC results at small $$r_s$$. However, beyond $$r_s>3$$ our VDMC results are closer to the theory which contains both the charge and the spin fluctuations ($$G_+ \& G_-$$), hence we can infer that at moderate correlations strength, the spin fluctuations start to play an important role, and charge fluctuations are no longer sufficient in determining the mass of the electron gas.

### The Landau liquid parameters

With precisely calculated effective mass, as well as the spin and charge susceptibility determined in our previous work^15^, we can calculate Landau parameters $F_0^a$ and $F_0^s$, which are obtained from $\frac{\chi _s}{\chi _s^0} = \frac{m^*}{m}\frac{1}{1+F_0^a}$ and $\frac{P_{q=0}}{P_{q=0}^0} = \frac{m^*}{m}\frac{1}{1+F_0^s}$ . Here $\chi _s$ and $P_q$ are the spin susceptibility and charge polarization, while $\chi _s^0$ and $P_q^0$ are their non-interacting analogues. In Table 1 we list our calculated Landau parameters $F^a_0$ and $F^s_0$, together with the estimation of their error, which mostly comes from error in determining spin and charge susceptibility in Ref. ^15^. While the Landau parameters, which determine the interaction between quasiparticle, have been estimated by various approximate numerical methods before^7^, to our knowledge their numerically controlled value has not be obtained before.

### The spectral function and the bandwidth

**Figure 4:**
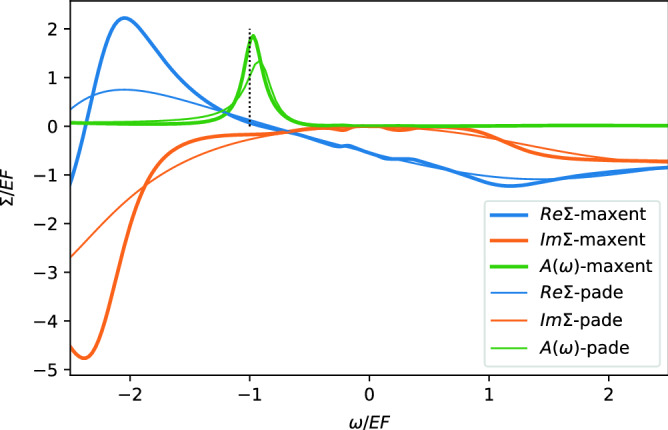
The spectral function and $\Sigma _{k=0}(\omega )$ at $r_s=4$ and $k=0$ as relevant for bandwidth of Na metal. The vertical dotted line marks the peak position of the non-interacting model. The thick and thin lines correspond to two different methods of analytical continuation, the maximum-entropy and Pade method, respectively.

The present VDMC algorithm also allows us to compute a numerically controlled value for the dynamic self-energy on the imaginary axis. Analytic continuation is needed to obtain the self-energy on the real frequency axis. In contrast to physical quantities computed from the imaginary axis data, the analytic continuation is not a numerically controlled method in which precise error bars would be available. We use the maximum entropy as well as the Pade method, to compute the quasiparticle energy at the $k=0$ point, which determines the bandwidth of the electron dispersion, i.e., the energy difference between the Fermi level and the lowest possible quasiparticle energy. The difference between these two standard analytic continuation methods gives a rough estimate of the error bar for the bandwidth. In Fig. 4 we display the self-energy, as well as the spectral function at momentum $k=0$ and finite frequency. We notice that the imaginary part of the self-energy starts to grow rapidly when the energy of the single-particle excitations exceeds the plasma frequency $\omega _p\approx 1.881 E_F$. Consequently, there appears a strong pole at $\omega < - 2 E_F$ due to such plasma excitations, and makes quasiparticle approximation invalid at a frequency below $\omega < - E_F$, as the real part of the self-energy is no longer a linear function of frequency. However, around $E_F$ the real-part of $\Sigma$ is still quite close to a linear function, and only minor deviations are noticed. Consequently, the renormalization of the dispersion can not substantially deviate from our earlier estimation of $m^*/m$, which is valid at the Fermi level. Our numerical estimation based on the analytically continued self-energy is that the spectral function at $r_s=4$ and $k=0$ has a peak around $- 0.96 E_F$ (maximum-entropy method) $- 0.93 E_F$ (Pade method), which deviates from the non-interacting value by 4–7%, hence the bandwidth reduction due to interactions at $r_s=4$ is approximately 4–7%. This value is much smaller than the experimental estimation of the bandwidth reduction in Na metal, in which the measured ARPES bandwidth appears to be renormalized for about 18–25%^32,33^. However, our estimated bandwidth is definitely not substantially larger as compared to the non-interacting bandwidth, in contrast to several other many-body calculations^5,34^, and is neither substantially smaller as in early GW calculations^35^ or GW with paramagnon vertex corrections^8^. Based on our very precise estimation of the single-particle self-energy, we can confidently exclude a possibility of such a dramatic reduction of the bandwidth in the model of electron gas due to correlation effects at the density corresponding to Na metal. This large reduction of the effective mass in ARPES thus requires an alternative explanation, which was assigned to the interaction in the final states^5,36^ in ARPES, surface effects^37^, and possibly the lattice effects, i.e, deviation of Na metal from the continuous model of the uniform electron gas.

In summary, we established the low energy excitation spectrum of the uniform electron gas at metallic density using recently developed VDMC. Controlled values of *Z*, $m^*/m$, $F_0^s$, and $F_0^a$ are given, which agree with the state of the art calculations in the field, but here we provide much more precise values than previously known.

## Methods

The Hamiltonian of UEG problem is

2 $$\begin{aligned} {\hat{H}}=\sum _{{\mathbf {k}}\sigma } \left( {{\mathbf {k}}^2}-\mu \right) {\hat{\psi }}^\dagger _{{\mathbf {k}}\sigma }{\hat{\psi }}_{{\mathbf {k}}\sigma }+\frac{1}{2V}\sum _{\begin{array}{c} {\mathbf {q}}\ne 0\\ {\mathbf {k}}{\mathbf {k}}'\sigma \sigma ' \end{array}} \frac{8\pi }{q^2}{\hat{\psi }}^\dagger _{{\mathbf {k}}+{\mathbf {q}}\sigma }{\hat{\psi }}^\dagger _{{\mathbf {k}}'-{\mathbf {q}}\sigma '}{\hat{\psi }}_{{\mathbf {k}}'\sigma '}{\hat{\psi }}_{{\mathbf {k}}\sigma }, \end{aligned}$$

where ${\hat{\psi }}$/${\hat{\psi }}^\dagger$ are the annihilation/creation operator of an electron, $\mu$ is the chemical potential controlling the density of the electrons in the system, and the long-range Coulomb repulsion is $8\pi /q^2$, as we measure the energy in units of Rydbergs, and the wave number *k*, *q* in units of inverse Bohr radius.

The expansion in terms of the bare interaction is divergent, therefore we first transform the original problem into an equivalent but a more appropriate problem for power expansion, which describes the emergent degrees of freedom at the lowest order, and the corrections are perturbatively included with very rapid convergence. Motivated by the well-known fact that the long-range Coulomb interaction is screened in the solid and that the effective potential of emerging quasiparticles differs from the bare potential, we introduce the screening parameter $\lambda _{\mathbf {q}}$ and an electron potential $v_{\mathbf {k}}$ into the quadratic part of the emergent Lagrangian $L_0$ of the form

3 $$\begin{aligned} L_0 = \sum _{{\mathbf {k}}\sigma }\psi ^\dagger _{{\mathbf {k}}\sigma } \left( \frac{\partial }{\partial \tau }-\mu +{\mathbf {k}}^2+v_{\mathbf {k}}(\xi =1)\right) \psi _{{\mathbf {k}}\sigma } +\sum _{{\mathbf {q}}\ne 0} \phi _{-{\mathbf {q}}} \frac{q^2+\lambda _{\mathbf {q}}}{8\pi }\phi _{\mathbf {q}}, . \end{aligned}$$

We then add the following interacting part to the Lagrangian

4 $$\begin{aligned} \Delta L =-\sum _{{\mathbf {k}}\sigma }\psi ^\dagger _{{\mathbf {k}}\sigma } v_{\mathbf {k}}(\xi )\psi _{{\mathbf {k}}\sigma } -\xi \sum _{{\mathbf {q}}\ne 0}\phi _{-{\mathbf {q}}} \frac{\lambda _{\mathbf {q}}}{8\pi }\phi _{\mathbf {q}}+\sqrt{\xi }\frac{i}{\sqrt{2 V}}\sum _{{\mathbf {q}}\ne 0} \left( \phi _{{\mathbf {q}}}\rho _{-{\mathbf {q}}}+\rho _{{\mathbf {q}}}\phi _{-{\mathbf {q}}}\right) . \end{aligned}$$

so that, when the number $\xi$ is set to unity, $L(\xi ) = L_0(\xi )+\Delta L(\xi )$ is Lagrangian of UEG. Indeed integrating out the bosonic fields $\phi _{\mathbf {q}}$ from Lagrangian *L*, we get the Lagrangian corresponding to the original Hamiltonian Eq. (2). Here $\rho _{\mathbf {q}}$ is the density fluctuation of the problem $\rho _{\mathbf {q}}=\sum _{{\mathbf {k}}\sigma }\psi ^\dagger _{{\mathbf {k}}\sigma }\psi _{{\mathbf {k}}+q\sigma }$. Note that the first two terms in $\Delta L$ are the counterterms^38^ which exactly cancel the two terms we added to $L_0$ above. We use the number $\xi$ to track the order of the Feynman diagrams so that order *N* contribution sums up all diagrams carrying the factor $\xi ^N$. We set $\xi =1$ once we enumerate all the diagrams of a certain order.

The emergent screening length $\lambda _{\mathbf {q}}$ and effective potential $v_{\mathbf {k}}$ are not a-priory known and need to be properly optimized to achieve an optimal speed of convergence. We note in passing that determining those parameters self-consistently, i.e., $\lambda _{\mathbf {q}}$ from the self-consistent polarization, and $v_{\mathbf {k}}$ from the single-particle self-energy, is not the most optimal choice for the speed of convergence. Determining them by the principal of minimal sensitivity is a much better choice, as pointed out by Kleinert and Feynman^39–42^. They showed that when an effective parameter of a theory is optimized with this principle, the perturbative expansion converges very fast, and can succeed even when the interaction is strong, and regular perturbation theory fails.

To make algorithm sufficiently simple to implement, we take $\lambda _{\mathbf {q}}$ to be ${\mathbf {q}}$ independent constant ($\lambda$), which is already sufficient for rapid convergence of the series. We emphasize that for any choice of these parameters we are guaranteed to converge to the same answer, provided that the series converges. Furthermore, we found that the convergence of the expansion is best when the Fermi surface of both the dressed $G_{\mathbf {k}}$ and the bare $G^0_{\mathbf {k}}$ Green’s function at each order is fixed with the Luttinger’s theorem so that the density and the Fermi surface volume is not changed with the increasing perturbation order. We therefore, expand $v_{\mathbf {k}}$ in power series $v_{\mathbf {k}}= (\Sigma ^x_{\mathbf {k}}(\lambda )-\Sigma ^x_{k_F}(\lambda )) + \xi \, s_1 + \xi ^2\, s_2\cdots$, and we determine $s_N$ so that all contributions at the order $\xi ^N$ do not alter the physical volume of the Fermi surface. Similarly to optimizing $\lambda _{\mathbf {q}}$, we found that it is sufficient to take $s_N$ constants independent of the momentum. Since the exchange ($\Sigma ^x_{\mathbf {k}}$) is static and is typically large, we accommodate it at the zeroth-order into the effective potential, so that at the first order we recover the GW type self-energy with $G_{\mathbf {k}}$ at the screened Hartree-Fock (screened by screening length $\lambda$) and exact $W_{\mathbf {q}}$.

As mentioned before, the algorithm depicted in Fig. 1 needs a numerically exact (converged) $W_{\mathbf {q}}$, which is first computed with the algorithm of Ref. ^15^. It was shown in Ref. ^15^ that the most rapidly converging scheme for charge and spin-susceptibilities is the so-called vertex correction scheme, in which we add an infinite sum of ladder diagrams on both sides of a polarization Feynman diagram. To do that, we first precompute the three-point ladder vertex and then attach it to both sides of a polarization Feynman diagram while the diagrams are sampled, and at the same time, we eliminate all ladder-type diagrams from the sampling, to avoid double-counting of diagrams. Next, we use Hedin’s type equation depicted in Fig. 1 in which one fermion propagator is dressed and requires self-consistent *G*. It easy to see that it is sufficient to use bold *G* of the lower order $N-1$ when evaluating self-energy at order *N*, to avoid the expensive self-consistent calculation. Finally, we use the finite temperature imaginary-time formalism, and we set the temperature to $T=0.04 \,E_F$, which is sufficiently below the Fermi liquid scale, so that is essentially equivalent to zero temperature.

## Note added

During the refereeing of this article, an alternative method based on the diffusion Monte Carlo (using fixed node approximation) has been used to compute the effective mass of the same model in Refs. ^43^. Their results are substantially different from ours, and show monotonically decreasing effective mass with increasing correlation strength (increasing $r_s$) reaching $m^*/m=0.85$ at $r_s\approx 4$, which is consistent with previous fixed node approximation work on Na metal^34^, that showed a considerable increase of the bandwidth in Na metal as compared to the LDA, in stark contrast to our results and even larger deviation from ARPES experiments of Refs. ^32,33^. In the MC work on Na metal^34^ the increase of the bandwidth was ascribed to the fixed node approximation, in which the quality of the nodal surface deteriorates at the bottom of the band, and hence leads to systematic error. An even more problematic issue in extracting the effective mass using diffusion Monte Carlo method was explained in Ref. ^44^ (see discussion around Fig. 23.3). Namely, the diffusion MC method uses a finite-size system simulations (in contrast to our method defined in the thermodynamic limit), in which the momentum resolution near the Fermi wave vector is limited, hence some further approximate assumptions are needed to extract the effective mass on the Fermi surface. This issue led to two very different extractions of the effective mass in the 2D electron gas (compare Ref. ^45^ and Ref. ^46^) using almost identical Monte Carlo data. In light of our controlled results for the effective mass of the 3D electron gas, it would be desired to revisit the analysis of variational MC data using the method of Ref. ^46^, which uses only the excitations in a narrow window of the Fermi level when extracting the effective mass, which would hopefully, be more consistent with our data and those of analytical theories which include charge and spin vertex corrections to GW^26^.

## Data Availability

The source code has been made available for download under gnu license at: https://github.com/haulek/VDMC.
